# Clinical Significance of Isolated Sphenoid Sinusitis Identified in Pediatric Patients Presenting with Headache

**DOI:** 10.3390/medicina60101625

**Published:** 2024-10-04

**Authors:** Seung Beom Han, Jee Min Kim, Eu Gene Park, Ji Yoon Han, Jin Lee

**Affiliations:** 1 Department of Pediatrics, College of Medicine, The Catholic University of Korea, Seoul 06591, Republic of Korea; beomsid@catholic.ac.kr (S.B.H.); eugene.park@catholic.ac.kr (E.G.P.); han024@catholic.ac.kr (J.Y.H.); 2 Department of Pediatrics, Seoul National University Children’s Hospital, Seoul 03080, Republic of Korea; 7a935@snuh.org

**Keywords:** headache, sinusitis, sphenoid sinusitis, child

## Abstract

*Background and Objectives*: Brain imaging studies in pediatric patients with headaches often reveal inflammation of the sphenoid sinus. When we encounter patients presenting with headaches without respiratory symptoms, determining the causal relationship between isolated sphenoid sinusitis observed in brain imaging studies and headache is challenging. This study aims to evaluate the clinical characteristics of pediatric patients with headaches and isolated sphenoid sinusitis identified by brain imaging studies and to determine the effects of antibiotics on headache relief. *Materials and Methods*: Among patients aged <18 years with headaches, those in whom isolated sphenoid sinusitis was observed on brain imaging were included. Their medical records were retrospectively reviewed to evaluate their clinical characteristics and outcomes. Based on antibiotic and analgesic effects, the included patients were categorized into acute bacterial sinusitis (BS) and non-BS groups, and clinical data were compared between the two groups. *Results*: Brain imaging studies were performed for 1751 patients, and 205 (11.7%) and 41 (2.3%) patients demonstrated sinusitis and isolated sphenoid sinusitis, respectively. For the 41 patients with isolated sphenoid sinusitis, migraine with or without aura (58.5%) was the most frequent type of headache. Throbbing pain (34.1%) occurred most frequently, and the temporal area (51.2%) was the most common location of headache. Nausea/vomiting (56.1%) was the most common accompanying symptom, followed by ocular symptoms (34.1%). Only one (2.4%) patient complained of neurologic symptoms. Headache improved in 26 (63.4%) patients, with improvement without antibiotic therapy in 19 (46.3%) patients. The acute BS and non-BS groups demonstrated comparable characteristics, except for a higher frequency of ocular symptoms in the acute BS group than in the non-BS group (*p* = 0.044). *Conclusions*: Isolated sphenoid sinusitis was rarely identified in pediatric patients with headache examined using brain imaging studies. Considering the clinical characteristics and antibiotic effects, early intensive antibiotic therapy cannot be prioritized.

## 1. Introduction

Headache is a prevalent issue among children and adolescents, with approximately 58% of them experiencing it [1]. Headaches are categorized into primary and secondary headaches based on the underlying cause [1]. The majority of cases of pediatric headaches are primary headaches, with migraine being the most common [1]. However, the evaluation of secondary headaches resulting from other pathologies is sometimes required [1]. Brain imaging studies are recommended for the evaluation of secondary headaches, especially for those accompanied by abnormal neurological examination results, seizures, underlying systemic disorders, and awakening from sleep due to headache [1]. In brain imaging studies conducted to evaluate secondary headaches in pediatric patients, inflammation of the paranasal sinuses is often detected. In the International Classification of Headache Disorders, 3rd edition, beta version (ICHD-3 beta), headache caused by sinusitis is classified as headache attributed to acute, chronic, or recurring rhinosinusitis [2]. To diagnose this particular type of secondary headache, clinical, endoscopic, and/or imaging evidence of sinusitis should be accompanied by at least two of the following criteria: (1) a temporal relationship between headache and sinusitis, (2) the concurrent worsening and/or improvement of headache and sinusitis symptoms, (3) headache exacerbation when placing pressure over the sinuses, and (4) ipsilateral headache consistent with sinusitis [2]. Among the paranasal sinuses, the sphenoid sinus is located deep centrally at the base of the skull. Therefore, evaluating the aggravation of headaches due to external pressure or ipsilateral headaches caused by sphenoid sinusitis can be challenging. Consequently, the causal relationship between sphenoid sinusitis observed in brain imaging studies and headache can be determined only based on coinciding changes in headache and sinusitis symptoms.

Among the various causes of sinusitis, acute bacterial sinusitis (BS) needs to be resolved with antibiotic therapy for more than 10 days, in contrast to sinusitis caused by viral infection or allergic rhinitis [3]. Considering that inappropriately prolonged antibiotic use is the principal inducer of antibiotic resistance, which is one of the top global public health concerns [4], the differential diagnosis of acute BS among sphenoid sinusitis cases observed in brain imaging studies is essential for appropriate antibiotic use in real-life clinical settings. Acute BS is diagnosed when persistent (>10 days) or worsening nasal discharge and/or a daytime cough, or concurrent purulent nasal discharge and fever for ≥3 days, are observed [3]. However, we encountered pediatric patients presenting with headaches without respiratory symptoms, in whom sphenoid sinusitis was observed in brain imaging studies; in addition, headaches improved with antibiotic therapy in some patients. In previous studies, nasal symptoms were observed in approximately 20% of pediatric patients diagnosed with acute bacterial sphenoid sinusitis [5], and in certain patients presenting with headaches, sphenoid sinusitis was diagnosed only after the occurrence of severe complications such as cranial nerve palsy, blindness, cavernous sinus thrombosis, stroke, and epidural abscess [6]. Therefore, diagnosing headache attributed to sphenoid sinusitis based solely on respiratory symptoms delays the diagnosis and treatment of acute bacterial sphenoid sinusitis, potentially leading to more complications.

This study aimed to evaluate the clinical characteristics of pediatric patients presenting with headaches in whom sphenoid sinusitis was identified in brain imaging studies; this was in order to identify predictive factors for acute BS in addition to respiratory symptoms and to evaluate the effects of antibiotic therapy on headache relief to determine the clinical significance of BS. The study results can provide information on the clinical significance of sphenoid sinusitis incidentally identified in pediatric patients with headaches and the necessity of antibiotic therapy for these patients.

## 2. Materials and Methods

### 2.1. Subject and Study Design

Pediatric patients aged <18 years who visited two university-affiliated hospitals (Incheon St. Mary’s Hospital [Incheon, Republic of Korea; Hospital A] and Daejeon St. Mary’s Hospital [Daejeon, Republic of Korea; Hospital B]) between January 2014 and December 2023 with a primary complaint of headache were considered for this retrospective cross-sectional observational study. Among them, those in whom brain imaging studies (computed tomography [CT] or magnetic resonance imaging [MRI]) for the evaluation of secondary headache were performed were selected. The brain imaging studies and modality selected were based on the discretion of the attending pediatric neurologist. The results of the brain imaging studies were retrospectively reviewed, and patients in whom sphenoid sinusitis was observed were included in this study. Sphenoid sinusitis was diagnosed when sinus mucosal thickening ≥ 3 mm, total opacification, or air–fluid level was observed in the sphenoid sinus [7]. The following patients were excluded from the study analysis: (1) those with underlying systemic disorders, (2) those with intracranial lesions, and (3) those with a history of head trauma within 3 months of brain imaging. Furthermore, when inflammation was observed in sinuses other than the sphenoid sinus, the patient was excluded, and only patients with isolated sphenoid sinusitis were included in the analysis. The medical records of the included patients were retrospectively reviewed to collect demographic data (sex and age) and clinical data. This included the type of headache; the characteristics, duration, accompanying symptoms, and patient’s family history of headache; previous history of allergies; medications administered to control headache; administered antibiotics; surgical intervention; and clinical response to therapy. The type of headache was decided based on the ICHD-3 beta [2]. Migraine, the most common type of pediatric headache, lasts for <72 h [2], and acute BS symptoms usually improve within 72 h of antibiotic therapy [3]. Determining the analgesic (acetaminophen or non-steroidal anti-inflammatory drugs) or antibiotic effects on headache 72 h after treatment is ideal; however, the interval for follow-up visits varied for each patient. Therefore, therapeutic effects were determined based on symptom improvement at the 1st follow-up visit after treatment. Based on the therapeutic effects, patients whose headaches improved after antibiotic therapy or surgical intervention for sinusitis were categorized into the acute BS group. If the headache improved without antibiotic therapy or if it did not improve even after appropriate antibiotic therapy, the patient was categorized into the non-BS group. The investigated demographic and clinical data were compared between the two groups. Patients whose headaches did not improve or those whose headaches improved with concurrent antibiotic and analgesic therapies were not categorized into either group. Isolated sphenoid sinusitis identified by brain imaging studies may be chronic BS accompanying a prolonged duration of headache or may be acute BS superimposed on a prolonged duration of headache. Although we could not clinically differentiate between these two situations, antibiotic therapy is not recommended for chronic BS because the effects of antibiotics on chronic BS have not been defined [8]. Because this study focused on the necessity of antibiotic therapy for isolated sphenoid sinusitis identified by brain imaging studies, the effects of antibiotics were determined based on acute BS.

### 2.2. Statistical Analysis

Descriptive statistics were analyzed for the entire study population. In the comparisons between the acute BS and non-BS groups, patient age was the only continuous factor, and it showed a normal distribution in the Kolmogorov–Smirnov test. Continuous factors were compared between the two groups using Student’s *t*-test, and categorical factors were compared using Fisher’s exact test. Statistical analyses were performed using the R Statistical Software (v4.3.3, R Core Team 2024, R Foundation for Statistical Computing, Vienna, Austria). Statistical significance was set at *p*-value < 0.05.

### 2.3. Ethics Statement

This study was approved by the Institutional Review Board of each hospital, which waived the requirement for informed consent (approval number: OC23RASI0159 for Hospital A and DC24RASI0047 for Hospital B).

## 3. Results

**Figure 1:**
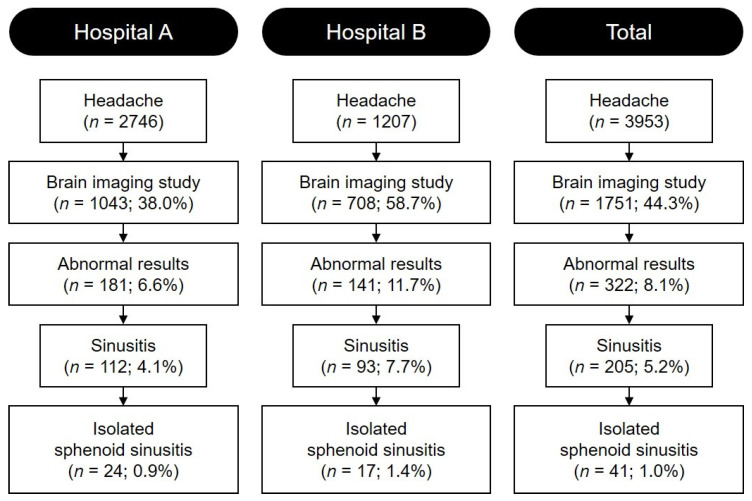
Flowchart for the study population.

During the study period at Hospital A, 2746 pediatric patients presented with headaches, and brain imaging studies were performed for 1043 (38.0%) patients (CT in 114 [10.9%] and MRI in 929 [89.1%]). Abnormalities were noted in 181 (17.4%) patients, with sinusitis identified in 112 (10.7%). Isolated sphenoid sinusitis was identified in 24 (2.3%) patients. In Hospital B, brain imaging studies were performed in 708 (58.7%; CT in 34 [4.8%] and MRI in 674 [95.2%]) of the 1207 pediatric patients presenting with headaches. Among them, 141 (19.9%) demonstrated abnormal results, with sinusitis and isolated sphenoid sinusitis identified in 93 (13.1%) and 17 (2.4%) patients, respectively. Brain imaging studies were performed in 44.3% (*n* = 1751) of 3953 pediatric patients presenting with headaches, with sinusitis identified in 5.2% (*n* = 205) and isolated sphenoid sinusitis in 1.0% (*n* = 41) of patients (Figure 1).

### 3.1. Characteristics of Pediatric Patients with Headache and Isolated Sphenoid Sinusitis

The 41 patients with isolated sphenoid sinusitis had a mean age of 9.9 ± 3.0 years, with 58.5% (*n* = 24) of them being men. The patients’ ages ranged from 5 to 17 years. The sphenoid sinus is observed in approximately 50% of 2-year-old children and in more than 90% of children older than 8 years, with expansion occurring between 3 and 8 years of age [7,9]. Therefore, all patients were considered to have significant sphenoid sinus inflammation and were included in the analysis. Sphenoid sinusitis was unilateral in 35 (85.4%; left in 20 and right in 15) and bilateral in six (14.6%) patients. No patient exhibited additional brain parenchymal abnormalities on imaging. Based on ICHD-3 beta, 24 (58.5%) patients were diagnosed with migraine with or without aura, and 11 (26.8%) were diagnosed with unspecified headache (Table 1). Secondary headache was diagnosed in three (7.3%) patients with sinusitis and one (2.4%) patient with a psychiatric disorder. Excluding five (12.2%) patients without a description of the headache location, 32 (78.0%) patients complained of localized headaches, while four (9.8%) reported diffuse headaches. For localized headache, the temporal area was the most frequent location, reported in 21 (65.6%; bi-temporal in 11, and uni-temporal in 10) patients, followed by the frontal area in eight (25.0%; bi-frontal in five, and uni-frontal in three), the vertex area in two (6.3%), the deep-seated area in two (6.3%), and the occipital area in one (3.1%) patient. Throbbing pain (*n* = 14, 34.1%) was the most common headache nature (Table 1). Most (*n* = 31, 75.6%) patients complained of moderate-intensity headaches, and the headache duration was less than 1 h in 46.3% (*n* = 19, Table 1). Nausea/vomiting (*n* = 23, 56.1%) was the most common accompanying symptom, followed by ocular symptoms including photophobia, blurred vision, diplopia, and ocular pain (*n* = 14, 34.1%), and dizziness (*n* = 13, 31.7%; Table 1). As a neurologic symptom, gait disturbance was observed in one (2.4%) patient. No patient complained of motor weakness, impairment of consciousness, dysesthesia, or seizures.

### 3.2. Clinical Outcomes and Comparison Results between the Acute BS and Non-BS Groups

Three (7.3%) patients were lost to follow-up after evaluation for headaches. Excluding these patients, headache improved in 26 (63.4%) of the remaining 38 (92.7%) patients at the follow-up visits. Antibiotics were administered in 12 (31.6%) patients; however, one (8.3%) patient received piperacillin, which is not recommended for treating acute BS. Therefore, this patient was excluded from the assessment of the antibiotic effects. Headache improved in two (16.7%) patients receiving antibiotic therapy without analgesics and in three (25.0%) patients receiving antibiotics and analgesics as needed. These five (41.7%) were included in the acute BS group. Analgesics administered as needed might not achieve a therapeutic steady-state concentration; therefore, their therapeutic effects were considered marginal. Another (8.3%) patient with headache improvement after receiving surgical intervention following failed antibiotic therapy was included in the acute BS group. The non-BS group included two (16.7%) patients without headache improvement after concurrent antibiotic and analgesic therapy and three (25.0%) patients who received antibiotic therapy after headache improvement. Of the 26 (68.4%) patients who did not receive antibiotic therapy, 10 (26.3%) did not experience headache improvement. The remaining 16 (42.1%) patients whose headaches improved without antibiotic therapy were included in the non-BS group. Six (15.8%) patients in the acute BS group and 21 (55.3%) in the non-BS group were comparatively analyzed (Table 2). Only one (16.7%) patient in the acute BS group exhibited respiratory symptoms consistent with acute BS. The acute BS and non-BS groups demonstrated comparable characteristics, except for a significantly higher frequency of ocular symptoms (*p* = 0.044) in the acute BS group than in the non-BS group (Table 2).

## 4. Discussion

In this study, the characteristics of headache in pediatric patients with isolated sphenoid sinusitis identified by brain imaging were comparable to the general characteristics of pediatric headache. Isolated sphenoid sinusitis was identified in 1.0% of pediatric patients who primarily presented with headache and in 2.3% of those who underwent brain imaging studies. Approximately 50% of the patients with isolated sphenoid sinusitis experienced headache improvement without antibiotic therapy.

The mean age of the patients included in this study was 9.9 years, with a predominance of males over females, consistent with previous reports demonstrating a higher prevalence of males among pre-adolescent school-aged children [1]. Consistent with the general characteristics of pediatric headache, migraine was the most common diagnosis. Throbbing pain in the bilateral temporal areas with moderate-to-severe intensity was the most commonly reported characteristics, similar to the features of pediatric migraine previously demonstrated [1]. Previous studies have noted challenges in differentiating between primary headaches and headaches attributed to sinusitis based solely on the clinical characteristics of the headache; therefore, 40% of pediatric patients with migraine and 60% of those with tension-type headaches were misdiagnosed with headaches attributed to sinusitis [10]. Considering that patients with isolated sphenoid sinusitis exhibit characteristics of general pediatric headache in this study, isolated sphenoid sinusitis identified on brain imaging studies may have little clinical impact and may not require antibiotic therapy. However, the sphenoid sinus, located deep within the central skull, is adjacent to several important structures, including cranial nerves III, IV, V1, V2, and VI, the optic nerve and chiasm, the internal carotid artery, the cavernous sinus, and the pituitary gland [11]. In the case of delayed treatment, bacterial sphenoid sinusitis may invade these structures, resulting in severe complications [11]. Therefore, the possibility of true bacterial sphenoid sinusitis identified by brain imaging studies cannot be ignored. As the pathognomonic characteristics of headache predicting acute BS have not been defined in previous studies on patients with sphenoid sinusitis who experienced headache improvement after antibiotic or surgical therapy [12], further investigations are required to identify the clinical factors predicting acute BS requiring antibiotic therapy in patients with isolated sphenoid sinusitis.

In a systematic review of 71 pediatric patients with acute bacterial sphenoid sinusitis, headache (98.6%) was the most common presenting symptom [5]. Nasal symptoms occurred in 22.5% of these patients, similar to our patients. Therefore, the presence of respiratory symptoms consistent with a diagnosis of acute BS may not be useful for deciding on antibiotic therapy in real-life clinical settings. In a previous study, 50.7% and 19.7% of patients complained of fever and ocular symptoms, respectively [5]. Meanwhile, in the acute BS group in this study, no patient complained of fever, and approximately two-thirds of the patients complained of ocular symptoms. In a previous study, patients visited hospital a median of 5.5 days (range: 1–90 days) after headache development [5], whereas about half of our patients visited hospital ≥3 months after headache development. Patients in the acute BS group visited hospital 2 days to 1 year after the development of headaches. Surgical intervention was performed in 31.0% of the previously reported patients, meanwhile headache improved in only one (1.4%) of the 71 patients without antibiotic therapy [5]. Among our patients, only one patient received surgical intervention and 50% (19/38) of them improved without antibiotic therapy. These discrepancies in the clinical characteristics and outcomes between our patients and previously reported patients with bacterial sphenoid sinusitis may imply that most of our patients did not have acute bacterial sphenoid sinusitis [5]. Sinusitis findings on brain MRI can be observed in patients with viral upper respiratory infections or allergic rhinitis, even in asymptomatic children [13,14]. Sphenoid sinusitis was observed in 15% of asymptomatic children [14]. With the increasing use of CT and MRI, the diagnosis of sphenoid sinusitis has risen [15]. In Korea, the National Health Insurance system covers approximately 98% of residents [16], and the Korean government expanded the insurance coverage for brain MRI in patients with headaches in 2017 [17]. Consequently, the number of brain MRI studies for patients with headaches increased from 8000 to 80,000 between 2017 and 2020, and a further increase in low-value care utilization, which provides little clinical benefit, higher healthcare costs, and increased complications, is expected [17]. The ability to access brain imaging modalities in Korea has likely facilitated the identification of more cases of sphenoid sinusitis, including those not caused by bacterial infections. In this study, isolated sphenoid sinusitis was identified in 2.3% (41/1751) of the pediatric patients examined using brain imaging. Even if we categorized the 14 patients in whom the antibiotic effects could not be evaluated in the acute BS group under the assumption that they had acute bacterial sphenoid sinusitis, only 1.1% (20/1751) of the patients examined by brain imaging were suspected to have acute bacterial sphenoid sinusitis. This low frequency of acute bacterial sphenoid sinusitis reported in the pediatric patients examined using brain imaging studies suggests that early intensive antibiotic therapy is not always necessary when imaging studies reveal isolated sphenoid sinusitis. Patients with respiratory symptoms consistent with the diagnostic criteria for acute BS should be treated with antibiotics. Considering the previously reported clinical characteristics of pediatric patients with acute bacterial sphenoid sinusitis [5], febrile patients with a relatively short headache duration of less than 3 months can be considered candidates for early antibiotic therapy. For patients without fever and respiratory symptoms, antibiotic therapy may be considered if their headaches demonstrate no improvement after a thorough evaluation of other causes and analgesic therapy.

Acute BS should be differentiated from rhinosinusitis due to other causes such as virus infection, allergy, asthma, and even immune deficiencies [18,19]. The rigorous documentation of personal and family histories of allergic disorders, recent medication, exposure to noxious environmental chemicals or pollutants, the seasonality of symptoms, and symptom duration is necessary [18]. The presence of sneezing, nasal/ocular itching, an allergic trigger, seasonal symptoms, non-purulent nasal discharge, and respiratory symptoms for less than 2 weeks usually indicates a non-bacterial cause of rhinosinusitis [18]. However, most patients with headache and isolated sphenoid sinusitis identified by brain imaging studies did not complain of respiratory symptoms in this study; therefore, such a differentiation might not be applicable in real-life clinical settings. Although ocular symptoms occurred more frequently in the acute BS group than in the non-BS group in this study, they seem to have a marginal clinical impact, as photophobia is present in approximately 60% of pediatric patients with migraine [20]. Considering the age range of pediatric patients with isolated sphenoid sinusitis, nasal endoscopy can be used to identify purulent sinus drainage and performing bacterial cultures, helping diagnose acute bacterial sphenoid sinusitis [21,22].

This study had some limitations. The number of included patients was small; however, the low frequency (2.7%) of sphenoid sinusitis among patients with sinusitis should be considered [23]. Although little effort has been made to determine the effects of antibiotics in pediatric patients with headache and isolated sphenoid sinusitis in previous studies reporting the characteristics of patients with isolated sphenoid sinusitis, we attempted to determine the therapeutic effects of antibiotics in these patients. Due to the retrospective nature of this study, we may have missed some data on clinical symptoms other than headache. Information regarding the patients’ recent history of respiratory symptoms and diagnoses potentially causing sinus inflammation before the brain imaging studies were performed was not sufficient. Although repeated brain imaging studies and laboratory tests are limited in pediatric patients compared with adults, information on bacterial culture results for respiratory secretions and changes in imaging and blood test results before and after antibiotic therapy was lacking in this study. To overcome these limitations, more attention should be paid to the presence and improvement of respiratory symptoms, and systematic follow-up strategies for blood, brain imaging, and culture studies should be established when examining patients with headaches and sinusitis identified by brain imaging. We plan to conduct a prospective study by recruiting pediatricians and otolaryngologists from all eight hospitals of the Catholic Medical Center in Korea to include as many patients as possible. As this study included patients who primarily complained of headaches, those with primary symptoms of fever or respiratory symptoms and accompanying headaches were excluded. However, such patients probably received empirical antibiotic therapy rather than brain imaging based on the clinical guidelines for acute rhinosinusitis [3].

## 5. Conclusions

In conclusion, isolated sphenoid sinusitis was rarely identified in pediatric patients who presented with headaches and were examined using brain imaging studies. Considering the clinical outcomes and effects of antibiotics, symptomatic therapy for headaches and a thorough evaluation of causes other than acute BS can be considered before early intensive antibiotic therapy is employed in patients without respiratory symptoms consistent with acute BS.

## Figures and Tables

**Table 1:** Characteristics of pediatric patients with headache and isolated sphenoid sinusitis.

Factor	Number of Patients (%) (*n* = 41)
Age, year, mean ± SD	9.9 ± 3.0
Male sex	24 (58.5)
Season on diagnosis	
Spring	10 (24.4)
Summer	14 (34.2)
Autumn	6 (14.6)
Winter	11(26.8)
Type of headache	
Migraine without aura	18 (43.9)
Migraine with aura	6 (14.6)
Tension-type headache	2 (4.9)
Secondary headache	4 (9.8)
Unspecified headache	11 (26.8)
Onset of headache	
<3 months before	20 (48.8)
≥3 months before	21 (51.2)
Headache nature	
Throbbing	14 (34.1)
Pressing	9 (22.0)
Stabbing	6 (14.6)
Pricking	4 (9.8)
Dull	1 (2.4)
Undescribed	7 (17.1)
Headache location	
Focal	32 (78.0)
Diffuse	4 (9.8)
Undescribed	5 (12.2)
Headache intensity	
Mild	4 (9.8)
Moderate	31 (75.6)
Severe	6 (14.6)
Headache duration	
<10 min	5 (12.2)
10–60 min	14 (34.1)
>60 min	22 (53.7)
Morning headache	8 (19.5)
Headache disturbing sleep	4 (9.8)
Family history of headache	14 (34.1)
Allergy history	4 (9.8)
Accompanying symptoms	
Nausea/vomiting	23 (56.1)
Ocular symptoms	14 (34.1)
Dizziness	13 (31.7)
Respiratory symptoms	9 (22.0)
Otologic symptoms	6 (14.6)
Abdominal pain	4 (9.8)
Fever	2 (4.9)

SD, standard deviation.

**Table 2:** Comparison between the non-BS and acute BS groups.

Factor	Non-BS Group (*n* = 21)	Acute BS Group (*n* = 6)	*p*-Value
Age, year, mean ± SD	9.4 ± 2.6	9.8 ± 4.2	0.747
Sex			1.000
Male	14 (66.7)	4 (66.7)	
Female	7 (33.3)	2 (33.3)	
Season on diagnosis			0.658
Spring	7 (33.3)	2 (33.3)	
Summer	6 (28.6)	2 (33.3)	
Autumn	4 (19.0)	0 (0.0)	
Winter	4 (19.0)	2 (33.3)	
Type of headache			0.050
Migraine	11 (52.4)	2 (33.3)	
Tension-type headache	2 (9.5)	0 (0.0)	
Secondary headache	1 (4.8)	3 (50.0)	
Unspecified headache	7 (33.3)	1 (16.7)	
Onset of headache			0.385
<3 months before	9 (42.9)	4 (66.7)	
≥3 months before	12 (57.1)	2 (33.3)	
Headache nature			0.096
Throbbing	9 (42.9)	0 (0.0)	
Pressing	5 (23.8)	2 (33.3)	
Stabbing	3 (14.3)	2 (33.3)	
Pricking	0 (0.0)	1 (16.7)	
Undescribed	4 (19.0)	1 (16.7)	
Headache location			0.454
Focal	15 (71.4)	6 (100.0)	
Diffuse	1 (4.8)	0 (0.0)	
Undescribed	5 (23.8)	0 (0.0)	
Headache intensity			0.115
Mild	0 (0.0)	1 (16.7)	
Moderate	20 (95.2)	4 (66.6)	
Severe	1 (4.8)	1 (16.7)	
Headache duration			0.321
<10 min	2 (9.5)	1 (16.7)	
10–60 min	11 (52.4)	1 (16.7)	
>60 min	8 (38.1)	4 (66.6)	
Morning headache	2 (9.5)	1 (16.7)	0.545
Headache disturbing sleep	1 (4.8)	0 (0.0)	1.000
Family history of headache	7 (33.3)	1 (16.7)	0.633
Allergy history	3 (14.3)	1 (16.7)	1.000
Antihistamine administration	2 (9.5)	2 (33.3)	0.204
Accompanying symptoms			
Nausea/vomiting	13 (61.9)	4 (66.7)	1.000
Ocular symptoms	4 (19.0)	4 (66.7)	0.044
Dizziness	5 (23.8)	2 (33.3)	0.633
Respiratory symptoms			
Cough	1 (4.8)	0 (0.0)	1.000
Rhinorrhea	3 (14.3)	2 (33.3)	0.303
Nasal stuffiness	1 (4.8)	1 (16.7)	0.402
Sputum	2 (9.5)	1 (16.7)	0.545
Otologic symptoms	3 (14.3)	1 (16.7)	1.000
Abdominal pain	3 (14.3)	0 (0.0)	1.000
Fever	1 (4.8)	0 (0.0)	1.000

SD, standard deviation.

## Data Availability

The data are available upon reasonable request from the corresponding author.
